# A structural phylogenetic tree of Rad52 and its annealase superfamily

**DOI:** 10.1016/j.csbj.2024.12.012

**Published:** 2024-12-24

**Authors:** Ali Al-Fatlawi, Md. Ballal Hossen, Stella de Paula Lopes, A. Francis Stewart, Michael Schroeder

**Affiliations:** a Biotechnology Center (BIOTEC), Center for Molecular and Cellular Bioengineering, Technische Universität Dresden, Dresden, Germany; b Center for Scalable Data Analytics and Artificial Intelligence (ScaDS.AI), Dresden, Germany

**Keywords:** Rad52, SSAP, DNA repair, AlphaFold, Homology

## Abstract

Rad52, a highly conserved eukaryotic protein, plays a crucial role in DNA repair, particularly in double-strand break repair. Recent findings reveal that its distinct structural features, including a characteristic *β*-sheet and *β*-hairpin motif, are shared with the lambda phage single-strand annealing protein, Red*β*, and other prokaryotic single-strand annealing proteins (SSAPs), indicating a common superfamily. Our analysis of over 10,000 SSAPs across all domains of life supports this hypothesis, confirming the presence of the characteristic motif despite variations in size and composition. We found that archaea, representing only 1% of the studied proteins, exhibit most of these variations as reflected by their spread across the phylogenetic tree, whereas eukaryotes exhibit only Rad52. By examining four representative archaeal SSAPs, we elucidate the structural relationship between eukaryotic and bacterial SSAPs, highlighting differences in *β*-sheet size and *β*-hairpin complexity. Furthermore, we identify an archaeal SSAP with a predicted structure nearly identical to human Rad52. Together with a screen of over 100 million unannotated proteins for potential SSAP candidates, our computational analysis complements the existing sequence and structural evidence supporting orthology among five SSAP families: Rad52, Red*β*, RecT, Erf, and Sak3.

## Graphical abstract

**Figure:**
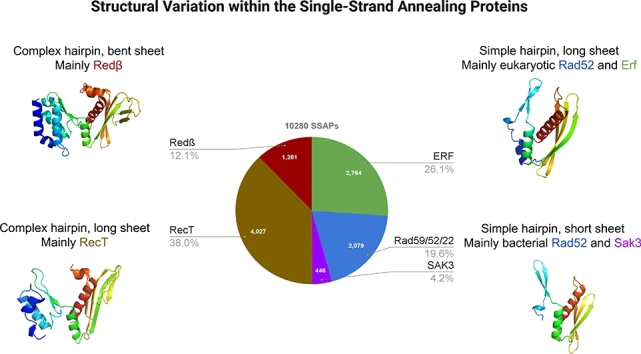

## 1 Introduction

Rad52 is a nearly ubiquitous eukaryotic protein involved in DNA repair, particularly in the repair of double-strand breaks by facilitating the pairing of complementary DNA strands [1], [2]. It functions in both homologous recombination (HR) and single-strand annealing (SSA) pathways and has been extensively studied in vitro for its ability to form undecameric rings in the absence of DNA [3], [4] and to promote annealing of complementary DNA strands [5]. These properties are also exhibited by the lambda phage single-strand annealing protein (SSAP), Red*β* [6]. Although other phage SSAPs form filaments rather than rings [7], Passy et al. [6] speculated that Rad52 and Red*β* are functionally related because they both form undecameric rings. However, Erler et al. [8] and Ander et al. [9] presented evidence that Red*β* promotes single-strand annealing by a monomer-to-multimer transition without the involvement of rings. Recently, Kharlamova et al. [10] provided evidence that Rad52 also facilitates single-strand annealing and homology detection primarily through monomer-to-multimer transitions rather than ring structures. The emergence of functional commonality in SSAP action strengthens the Rad52 superfamily hypothesis [8].

Using advanced bioinformatic tools, Erler et al. [8] identified a distant similarity between Rad52 and Red*β*, coinciding with the most conserved sequences within the Rad52 class, suggesting an orthologous relationship between these two distantly separated SSAPs.

Using advanced bioinformatics tools, Erler et al. [8] identified a distant tripartite amino acid signature as a similarity between Rad52 and Red*β*. Notably this tripartite signature coincided with the most conserved sequences within the Rad52 class, suggesting an orthologous relationship between these disparate SSAPs. Several further observations further supported the idea that some, perhaps all, SSAPs were orthologous related: (i) all SSAP single-strand DNA binding/annealing domains are N-terminally located in the first approximately 180 amino acids, which includes the tripartite amino acid signature; (ii) in the absence of DNA, they multimerize into rings or chains at high concentrations in vitro (>0.5 M); (iii) they bind ssDNA with modest affinity but have low affinity for double-strand (ds) DNA; (iv) beyond the N-terminal annealing domain, the C-terminal regions are not required for annealing but for interactions with partner proteins that facilitate homologous recombination (HR) [11], [12]. Independently, the distant sequence relationship between Rad52 and the Red*β*/RecT classes was confirmed using different bioinformatic methodologies [13], and the Erf class was included in the proposed SSAP superfamily [14].

The Rad52 superfamily hypothesis was recently strengthened by the cryo-EM structural resolution of two members of the Red*β*/RecT class [15], [16], which identified structural similarities with the known Rad52 structure [3], [4]. Additionally, AlphaFold predictions revealed a new protein fold [17]. This fold, shared by the Erf class, includes a well-conserved three-stranded *β*-sheet traversed on the inside by an *α*-helix. The outside of the *β*-sheet determines the curvature of the helical filaments and rings formed by these SSAPs, while the traversing *α*-helix is stabilized by a second *α*-helix, an accompanying *β*-hairpin, and another *α*-helix. These latter secondary elements show considerable variability among various SSAPs. Previously, we [17] visually depicted the similarities and variations of this arrangement across eight selected SSAPs.

Here, we apply the discovery of the Rad52 superfamily and its unique protein fold across bacterial, archaeal, and eukaryotic proteomes. Following Illergard et al. [18], who argue that structure is three to ten times more conserved than sequence, we compare these SSAPs by their structure instead of their sequence. Such a large-scale comparison is made possible by the recent availability of high-quality predicted protein structures in the AlphaFold database [19].

We explored phylogenies computed from sequence and structure (Fig. S1), focusing on the preservation of the central *β*-hairpin and *β*-sheet with bridging *α*-helix motif across all superkingdoms. The majority of known SSAPs are bacterial and phage-derived, complemented by eukaryotic Rad52 proteins and a small number of archaeal sequences. We quantify variations in the core SSAP structural motifs across these distant relationships and exploit a structural perspective to screen for potential novel SSAPs among millions of unannotated proteins.

## 2 Results

### 2.1 10,280 SSAP predicted structures

Our study presents a comprehensive structural analysis of Rad52 and its annealase superfamily, providing insights into their structural diversity. We retrieved members of the single-strand annealing protein families—Rad59/52/22 (collectively referred to as Rad52 throughout this manuscript), Erf, Sak3, Red*β*, and RecT—from the InterPro database [20]. These datasets were enriched with 3D structures predicted by AlphaFold [19], [21]. After filtering out low-confidence structures (see Methods), we focused on 10,280 high-quality predicted protein structures: 5,150 from the Red*β* and RecT families, and a roughly equal number from Rad52, Erf, and Sak3.

### 2.2 Most SSAPs are bacterial and phage

Next, we enriched the data with phylogenetic information. An interesting hypothesis on the origin of eukaryotic Rad52 emerges from the highly imbalanced breakdown by superkingdoms (Table 1): 90% of the total SSAPs surveyed are of bacterial or phage origin. Given the frequency of horizontal gene transfer in bacteria, we consider bacterial and phage origins to be the same. The remaining 10% are split between eukaryotes (9%) and archaea (1%). While the 1% of archaeal predicted structures cover all five families, eukaryotic SSAPs are predominantly limited to Rad52. Notably, Rad52 is also present in bacteria and archaea. Our data suggest that the full diversity of SSAP families possibly originated in bacteria and archaea, with Rad52 being selected for in eukaryotes. Subsequent analysis explores how these evolutionary relationships manifest in the structural characteristics of SSAPs.

**Table 1:** Distribution of Rad52, RecT, Red*β*, Erf, and Sak3 Families Across Archaea, Eukaryota, and Bacteria.

Family	Archaea	Eukaryota	Bacteria	Total
Rad52	13	854	1,178	2,045
RecT	25	5	3,860	3,890
Red*β*	8	0	1,252	1,260
Erf	63	2	2,582	2,647
Sak3	15	0	423	438

**Total**	124	861	9,295	10,280

### 2.3 Predicted structure and sequence phylogenetic trees largely agree:

Protein families within the InterPro database are delineated based on sequence data using sophisticated algorithms like hidden Markov models. We compared 10,280 SSAPs using both sequence alignment (via the BLAST algorithm) and structural alignment (via the TM-align algorithm) to evaluate the consistency between structural and sequence-based classifications. The dendrograms generated from these comparisons (Fig. S1) clearly separate all five families, particularly distinguishing Rad52 and Red*β*. The clustering patterns consistently differentiate eukaryotic from bacterial Rad52. However, discrepancies arose in the placement of a Rad52 subgroup termed RDM, attributed to its additional RNA-binding motif. Despite this, our findings underscore the overall agreement between structural and sequence-based classifications of SSAPs.

### 2.4 Archaeal SSAPs represent all SSAP families

Among the SSAPs analyzed, less than 1% were found in archaea, a superkingdom known for harboring extremophiles. The question of how eukaryotes evolved from a world with only two domains, bacteria and archaea, remains open [22]. Given the evolutionary significance of archaea in understanding eukaryotic origins, we investigated potential structural distinctions among archaeal, bacterial, and eukaryotic SSAPs.

**Fig. 1:**
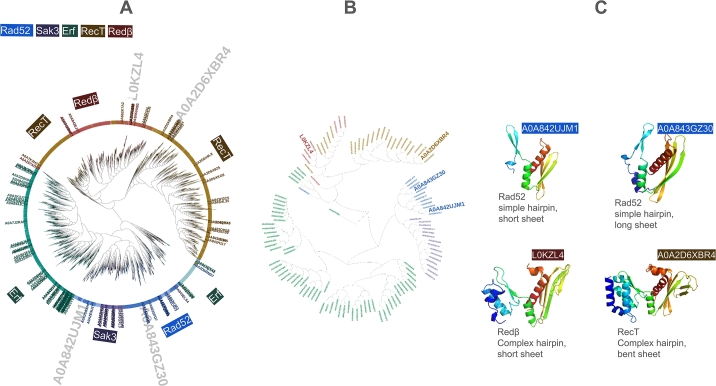
(A) 10,280 SSAPs clustered by structural similarity confirm the definition of SSAP families: RecT (brown), Red*β* (red), Erf (green), Sak3 (purple), and Rad52 (blue). RecT/Red*β* are clearly separated from Erf, Sak3, and Rad52. Sak3 is placed within the Rad52 cluster. Eukaryotic Rad52 (light blue) and prokaryotic Rad52 (dark blue) are resolved together, while the RDM1 subgroup (turquoise), which has an additional RNA-binding motif, is a sister clade. The 124 archaeal SSAPs are labeled by their IDs, showing that they cover the full diversity of the 10,280 SSAPs. (B) Close-up of the 124 archaeal SSAPs clustered by structural similarity, with the four selected representatives labeled by their identifiers. (C) The four selected representatives with their *β*-hairpin and *β*-sheet motifs, serving as references in subsequent analyses.

To determine if archaeal SSAP predicted structures differ significantly from bacterial and eukaryotic ones, we clustered all SSAPs by their structure and highlighted archaeal SSAPs (Fig. 1A). The 124 archaeal SSAPs are evenly distributed across the tree of 10,280 predicted structures, indicating they represent the full dataset. We selected 12 representative archaeal structures through clustering, refining them manually to four representative structures. These structures showcase variations in *β*-sheet and *β*-hairpin motifs, including strand length and hairpin complexity. Two representatives belong to the Rad52 family, encompassing Erf and Sak3, while the others represent RecT and Red*β*. This underscores the structural diversity within archaeal SSAPs.

### 2.5 Four representative SSAPs align with experimental templates

For the Rad52, RecT, and Red*β* families, which have experimentally determined 3D structures (from human and two bacterial phages), we assessed the alignment of the evolutionarily distant archaeal representatives. Using the TM-score (where values >0.5 indicate structural similarity), we obtained scores of 0.51 for Rad52, 0.65 for RecT, and 0.55 for Red*β*. In contrast, sequence identities on aligned motifs were notably low (5-8%). These findings highlight that, despite minimal sequence similarity, structural similarity is clearly evident.

These results refer to monomeric structures. However, SSAPs oligomerize to form regular quaternary structures, such as the Rad52 undecameric ring [4]. This raises the question of whether the predicted archaeal Rad52 representatives can form such ring structures. Neural networks for 3D structure prediction, such as AlphaFold, are trained on monomers, not oligomers. To investigate, we superimposed 11 copies of the predicted archaeal Rad52 structures onto the experimentally determined Rad52 undecameric ring template (Fig. S4 and Fig. S5).

To evaluate the feasibility of this hypothetical ring structure, we counted atom clashes between neighboring monomers. For the long *β*-sheet Rad52 representative, only 9 out of 869 atoms clashed at a 2Å distance cut-off. For the short *β*-sheet representative, the number of clashes was similarly low (8 out of 749 atoms). We performed similar analyses for Red*β* and RecT. Experimental structures for these two SSAPs form large helical structures rather than rings [15], [16]. The predicted archaeal Red*β* representative resulted in 16 clashes out of 1,087 atoms, while the RecT representative had 12 clashes out of 1,236 atoms. These results indicate that AlphaFold can accurately predict aspects of quaternary structure, even for oligomers.

### 2.6 Do Red*β* and RecT form a single group as suggested by Iyer et al. [23]?

**Fig. 2:**
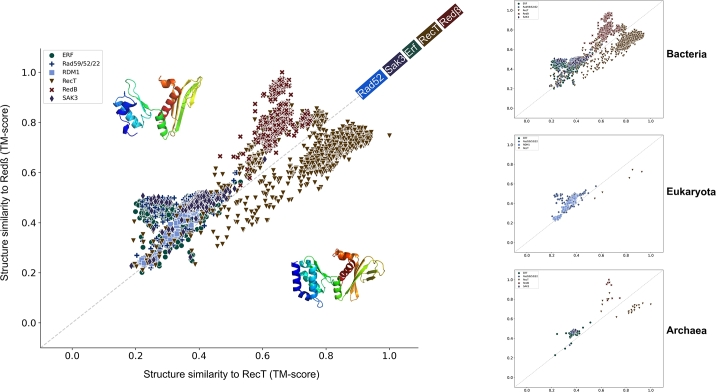
Scatter plot of structural similarity (TM-score) for each of the 10,280 SSAPs compared to the RecT (brown) and Red*β* (red) representatives. The RecT and Red*β* SSAPs are clearly distinct from other families and are also separated from each other. On the right, the three plots show the same settings as on the left, but for each superkingdom separately.

Using sequence analyses of SSAPs, Iyer et al. [23] proposed that RecT and Red*β* constitute a single family distinct from the Rad52 and Erf families. Our sequence and structure analyses confirm the distinction between RecT and Red*β*, although they exhibit closer structural similarity to each other than to the other families (Fig. 1A, Fig. S1A,B). To investigate these relationships further, we plotted the structural similarity of all SSAPs against the archaeal RecT and Red*β* representatives (Fig. 2). Structural similarity ranges from 0 (not similar) to 1 (identical), with values of 0.5 or higher indicating a similar fold [24], [25].

Nearly all RecT and Red*β* SSAPs exhibit a structural similarity of 0.5 or better to both RecT and Red*β* representatives. In contrast, the other three families—Rad52, Erf, and Sak3—consistently fall below 0.5 similarity to either of the RecT and Red*β* representatives. These findings support Iyer et al.'s [23] hypothesis. However, all Red*β* SSAPs are more similar to the Red*β* representative than to the RecT representative, and nearly all RecT SSAPs are more similar to the RecT representative than to the Red*β* representative. This clear separation supports the notion that RecT and Red*β* should be treated as distinct families, contrary to the proposal by Iyer et al. [23].

### 2.7 How do Red*β* and RecT compare to rad52, particularly eukaryotic Rad52?

**Fig. 3:**
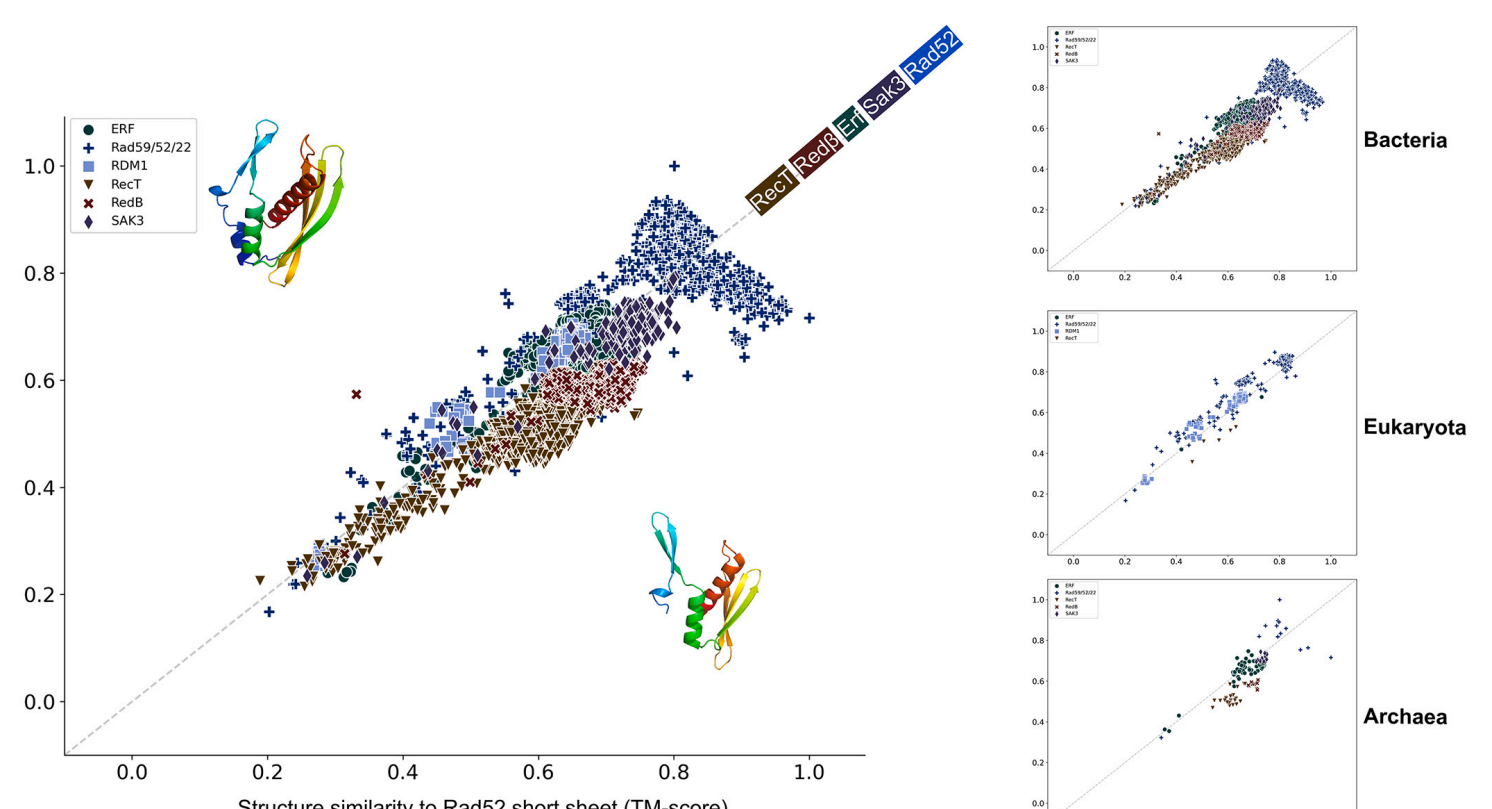
Scatter plot of structural similarity (TM-score) for each of the 10,280 SSAPs compared to the long and short *β*-sheet Rad52 representatives. Most SSAPs exhibit significant structural similarity. Bacterial Rad52 SSAPs (dark blue) are similar to both the long and short *β*-sheet Rad52 representatives. Eukaryotic Rad52 SSAPs (medium blue) are also highly similar to both but display greater similarity to the long *β*-sheet Rad52. Due to its additional RNA-binding domain, the Rad52 subfamily RDM1 (light blue) is more dissimilar to both representatives. On the right, the three plots show the same settings as on the left but are separated by superkingdom.

Iyer et al. [23] noted that RecT and Red*β* are distinct from the Rad52 and Erf families. This distinction is also supported by the clusterings in Fig. 1A and Fig. S1A,B. To explore these relationships in detail, we compared the structural similarity of all SSAPs against two representative archaeal Rad52 structures, which differ in the lengths of the three *β*-strands in the characteristic *β*-sheet motif (defined here as long and short *β*-sheets; Fig. 3).

In contrast to the previous analysis, all but a few RecT structures exhibit a structural similarity of 0.5 or better with both representatives, indicating similarity. As expected, Rad52, Erf, and Sak3 show higher similarity to these Rad52 representatives. Interestingly, nearly all Red*β*, RecT, and Sak3 SSAPs are more similar to the short *β*-sheet representative than to the long *β*-sheet representative. In contrast, bacterial Rad52 shows a split: 802 SSAPs (65%) are more similar to the long *β*-sheet representative, while 376 SSAPs (35%) are more similar to the short *β*-sheet representative. Eukaryotic Rad52 is predominantly similar to the long *β*-sheet representative.

Some of the Rad52 are characterized by poor similarity to either of the two Rad52 representatives. In fact, these SSAPs form a Rad52 subfamily called RDM1 (Rad52 Motif Containing 1) (Supplementary Fig. S6). RDM1, also termed RAD52 homologue B, contains a small RAD52-like (RD) motif shared with the recombination and repair protein RAD52, as well as an RNA Recognition Motif (RRM). Due to the presence of the RRM, RDM1 could bind to both RNA and DNA [26] suggesting roles in DNA repair and RNA metabolism and a function distinct from RAD52.

### 2.8 Do all SSAPs exhibit the characteristic motif?

Single-strand annealing proteins (SSAPs) are defined by their function, which is closely linked to the *β*-hairpin and *β*-sheet motifs that play a critical role in DNA binding. Do all 10,280 SSAPs contain these two motifs? As shown in Fig. 1C, these motifs are present in the four selected representatives. In this analysis, we computed the structural similarity (TM scores) for each of these 10,280 predicted structures against the four representative structures.

Of the 10,280 SSAPs analyzed, 208 have a TM-score below 0.5, indicating structural dissimilarity. Another 2,995 SSAPs fall within the TM-score range of 0.5 to 0.7, suggesting moderate structural similarity. Remarkably, the majority—7,077 SSAPs—demonstrate a TM-score above 0.7, indicating high structural similarity within this significant portion of the dataset (Supplementary Table S1). Visual inspection confirmed that a TM-score above 0.5 implies conservation of the *β*-sheet motif and the presence of the *β*-hairpin motif. A score above 0.7 indicates that both motifs are well conserved.

Overall, 98% of the SSAPs have a TM-score greater than 0.5 when compared to at least one of the four representatives (Table 2). For 69% of SSAPs, the TM-score exceeds 0.7. Additionally, 50% of the SSAPs have a TM-score greater than 0.5 when compared to at least three of the four representatives. From this, we conclude that the vast majority of SSAPs contain the two characteristic motifs, which are essential for single-strand annealing. This comprehensive analysis underscores the fundamental role of the *β*-hairpin and *β*-sheet motifs in SSAP function.

**Table 2:** Number of SSAPs with TM-scores greater than 0.5, 0.6, or 0.7 against 1, 2, 3, or 4 archaeal representative proteins. For example, in the first row, the reported values correspond to those with structural similarity above the threshold for at least one of the four representatives. In the second row, the values are for those meeting the threshold for at least two representatives, and so on.

Threshold	10,280 SSAPs			Unannotated Proteins		
	>0.5	>0.6	>0.7	>0.5	>0.6	>0.7
>0	10,072	9,947	7,077	206,859	8,136	3,143
>1	10,003	9,218	3,876	61,842	5,937	1,797
>2	5,146	3,011	3	8,148	763	0
>3	3,663	473	0	2,131	99	0

These results indicate that most SSAPs share significant structural similarity with at least one of the four representative predicted structures. The remaining 2%—208 proteins—primarily consist of fragments with an average length of approximately 160 amino acids, compared to 268 amino acids for the other SSAPs (Fig. S2). These fragments are likely incomplete sequences, which explains their lower TM-scores. Additionally, a small subset of full-length proteins with TM-scores around 0.4 exhibit divergent folds that lack the exact structural motifs characteristic of SSAPs. These cases may represent edge instances of the SSAP family or potential misannotations.

### 2.9 Quantifying motif alignment lengths across all families

To quantify the structural diversity and conservation of the *β*-hairpin and *β*-sheet motifs across the SSAP families, we employed two approaches. First, we analyzed the distribution of alignment lengths against the representative structures. Second, we quantified the composition of helix, strand, and loop residues in the motifs.

**Fig. 4:**
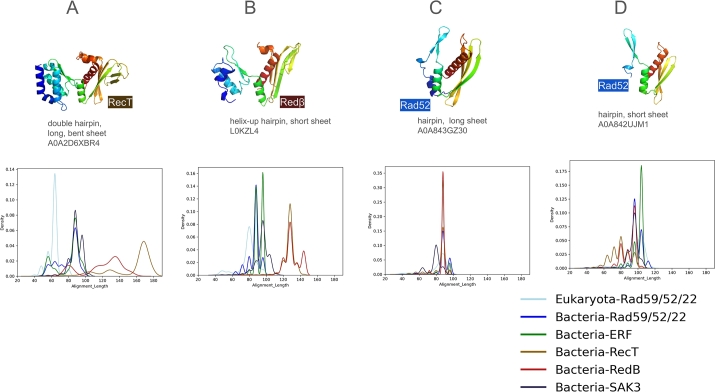
Distribution of alignment lengths (number of residues aligned) against four representative SSAPs. Structural alignment lengths are shown for: (A) RecT, (B) Red*β*, (C) Rad52 (long *β*-sheet), and (D) Rad52 (short *β*-sheet).

Fig. 1C visually illustrates the variation in size and shape of the *β*-hairpin and *β*-sheet motifs using four archaeal representatives. In Fig. 4, we show the alignment length of each of the 10,280 SSAPs, grouped by family and compared to each representative. Overall, alignments range from 60 to 180 residues in length.

The most pronounced peak is seen for the RecT family (Fig. 4A), with alignments clustering around 170 residues, confirming that RecT is structurally coherent and distinct from Red*β*. Fig. 4B shows a peak for Red*β* alignments, which range from 120 to 140 residues. Other bacterial SSAPs align with the RecT representative at lengths between 80 and 100 residues, while eukaryotic Rad52 shows alignments as short as 60 residues. When using Red*β* as the representative, a consistent pattern emerges: RecT and Red*β* are clearly separated from the other families. Most RecT and Red*β* proteins align with the Red*β* representative at around 130 residues, with a smaller subset aligning at 145 residues. Interestingly, eukaryotic Rad52 aligns better with Red*β* (peaking at 80 residues) than with RecT (60 residues).

For the Rad52 representatives (Fig. 4C,D), which feature long and short *β*-sheet motifs, notable differences are observed. In the short *β*-sheet motif (Fig. 4D), eukaryotic Rad52, bacterial Rad52, and the large Erf family align compactly at lengths over 100 residues. The similarity between Erf and Rad52 supports our choice of representatives, even though no Erf family members were selected as representatives. In Fig. 4C, the long *β*-sheet representative shows alignments across all families, including RecT, at reduced lengths of about 90 residues.

To further quantify motif variation, we analyzed the secondary structure composition, focusing on helix and strand lengths. This analysis included families and superkingdoms with more than 100 members (i.e., bacterial SSAPs and eukaryotic Rad52). The number of strand residues in the *β*-hairpin motif remains consistent across families and domains, ranging from 11 to 15 residues (Table 3).

**Table 3:** Number of helix (H) and strand (S) residues in the *β*-sheet and *β*-hairpin motifs for eukaryotic Rad52 and bacterial Rad52, Red*β*, RecT, Erf, and Sak3.

SS	SSAP	Superkingdom	*β*-Sheet Motif		*β*-Hairpin Motif	
			Median	Std	Median	Std
H	RecT	Bacteria	32	6.26	42	7.44
H	Red*β*	Bacteria	26	3.57	28	6.95
H	Erf	Bacteria	20	3.03	15	4.80
H	Rad52	Bacteria	17	2.87	12	3.76
H	Rad52	Eukaryota	15	4.50	18	7.42
H	Sak3	Bacteria	15	3.20	3	6.43

S	Rad52	Eukaryota	37	8.06	11	10.32
S	RecT	Bacteria	35	8.18	12	5.25
S	Red*β*	Bacteria	32	6.54	13	4.63
S	Erf	Bacteria	29	7.83	12	10.64
S	Rad52	Bacteria	27	7.69	14	5.66
S	Sak3	Bacteria	26	10.76	16	7.11

Our analysis supports the distinction between RecT and Red*β*, while also highlighting differences between these two families. Eukaryotic Rad52 can be distinguished from bacterial Rad52, which forms two groups: one similar to eukaryotic Rad52 and one distinct. Differences in helix residues are notable: Sak3 shows no helix in the hairpin motif (3 residues), whereas Red*β* and RecT exhibit pronounced helices (28 and 42 residues, respectively). This distinction is also evident in the *β*-sheet motif, where Rad52, Erf, and Sak3 have shorter helices (15-20 residues) compared to Red*β* and RecT (26 and 32 residues).

Strand lengths also vary: eukaryotic Rad52 has 37 residues compared to 27 residues in bacterial Rad52. RecT and Red*β* have strand lengths similar to eukaryotic Rad52, while Erf and Sak3 align more closely with bacterial Rad52. These findings highlight how sequence variations drive structural diversity while preserving the core motif topology.

### 2.10 Are there novel SSAPs?

The SSAPs documented in the InterPro database were assigned based on sequence information [20], [27]. Since structural information tends to be more conserved than sequence [18], we anticipated the existence of novel candidate SSAPs.

Some identified proteins were classified as RAD52-like by InterPro but were not included in our initial analysis of the 10,280 SSAPs. One example is the mitochondrial genome maintenance protein (Mgm101), an SSAP required for mitochondrial DNA (mtDNA) repair and maintenance. In our analysis, we found that 1,073 proteins exhibited significant structural similarity to the Rad52 long *β*-sheet motif, with TM-scores greater than 0.7.

**Fig. 5:**
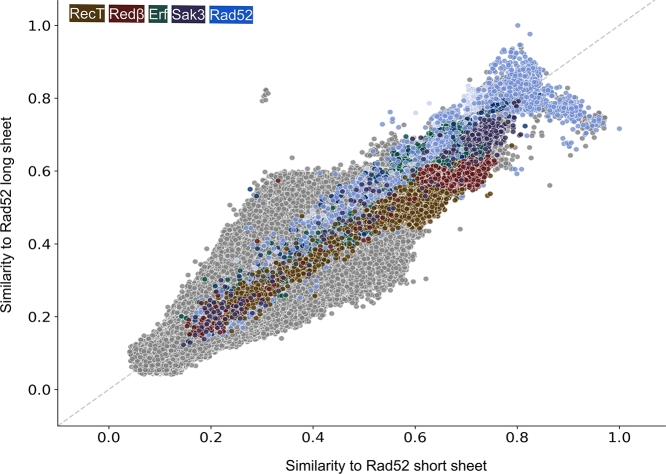
Scatter plot of structural similarity (TM-score) for all AlphaFold structures, including 117.5 million unreviewed proteins, compared to the two representative Rad52 SSAPs. The bulk of the proteins have TM-scores below 0.5. Many known SSAPs have TM-scores above 0.5. Among the top-scoring proteins, 2,164 (1,458) proteins have a TM-score greater than 0.7 against the long (short) *β*-sheet Rad52 representative. These are potential novel SSAP candidates.

We expanded our analysis to include 117,501,756 proteins listed in UniProt but not annotated by InterPro. Comparing these proteins to the four SSAP representatives, we found that 206,859 proteins exhibit structural similarity (TM-score greater than 0.5) to at least one representative. Applying a more stringent cut-off of 0.7, this number shrinks to 3,143 proteins. The majority show high similarity to the long (2,164 proteins) and short (1,458 proteins) *β*-sheet Rad52 representatives. A smaller subset shows similarity to RecT (667 proteins) and Red*β* (655 proteins) representatives (Fig. 5).

Computationally, many of these proteins are novel SSAP candidates. However, they remain unconfirmed until validated experimentally. This is often the case with structurally predicted proteins, where structural similarity suggests potential function but requires further verification. These results provide a solid foundation for subsequent experimental investigations to confirm their roles as novel SSAPs.

### 2.11 Oceanic archaeal SSAP resembles human Rad52

Remarkably, we identified an oceanic archaeal SSAP (UniProt ID: A0A2D6XHC3) that structurally resembles human Rad52 despite low amino acid sequence identity. This archaeal SSAP, derived from the Candidatus Pacearchaeota archaeon, was discovered in a metagenomic study conducted during the Tara Oceans circumnavigation expedition [28].

**Fig. 6:**
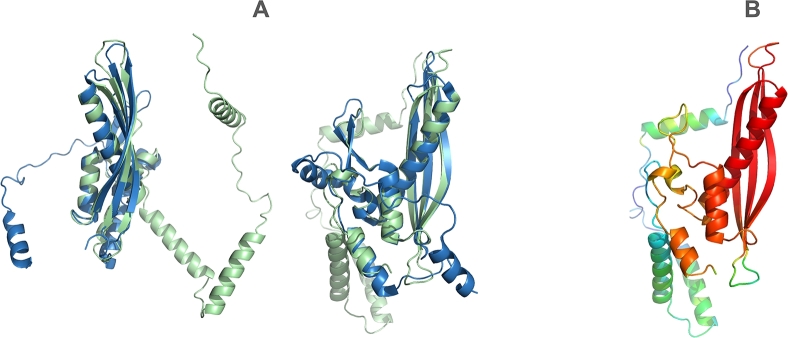
(A) Structural alignment of the archaeal SSAP A0A2D6XHC3 (green) with human Rad52 (blue) shown from different projection angles. The two structures align closely, with a TM-score of 0.82. (B) Confidence score (pLDDT) of the AlphaFold prediction. Red indicates very high confidence, while blue indicates low confidence. The discussed motif is highlighted in red, indicating a high-confidence prediction.

Although the sequence identity is only 30% at 47% coverage (effectively less than 15% sequence identity), the predicted archaeal structure and the experimentally determined human Rad52 structure exhibit remarkable structural similarity, with a TM-score of 0.82 (Fig. 6).

This finding highlights the remarkable conservation of structural features across evolutionarily distant organisms, suggesting functional conservation in their roles as single-strand annealing proteins. It underscores the importance of incorporating structural information alongside sequence data when studying protein evolution and function.

## 3 Discussion

### 3.1 SSAPs across all three superkingdoms

Despite sequence divergence, a common structural motif is shared by single-strand annealing proteins (SSAPs). Two key findings stand out: First, the few archaeal SSAPs exhibit the same structural diversity as the numerous bacterial SSAPs. Second, while all five SSAP families are present in archaea and bacteria, eukaryotes predominantly harbor the Rad52 family, with few exceptions.

Consistent with Williams et al. [22], who posit that archaea and bacteria are the two primary domains from which eukaryotes arose, we infer that the ancestral eukaryotic selection of Rad52 to the exclusion of other SSAPs has a functional basis. Eme et al. [29] also demonstrated that archaea carry several genes previously thought to be eukaryotic-specific. This aligns with our finding of significant structural similarity between eukaryotic and bacterial Rad52, which generally differ in the length of strands within the *β*-sheet motif (Fig. 3). Additionally, we identified an archaeal SSAP presenting a *β*-hairpin and *β*-sheet nearly identical to human RAD52. Together with the finding that only Rad52 is found in eukaryotes, this observation suggests that the Rad52 structural SSAP version conveys a specific functional variation distinct from the four other SSAP versions.

We were surprised to find that only 1% of SSAPs are archaeal. While this figure is consistent with the biomass estimate by Bar-On et al. [30], it contrasts with Karner et al. [31], who estimate that archaea and bacteria exist in similar magnitudes in oceanic environments. The discrepancy may arise from difficulties in relating biomass or cell numbers to sequence representation in databases. To address this, we counted archaeal sequences in UniProt and found 6,606,939 archaeal sequences out of 252,170,925 total sequences, representing 2.6%. Therefore, the 1% representation of archaeal SSAPs is only slightly lower than their overall representation in UniProt.

The advent of large-scale structure prediction has enabled the largest structural comparison of the Rad52 superfamily to date. Interestingly, predicted structures not only resemble the overall SSAP structural motif but also aspects of quaternary structure, such as the width and shape of SSAP monomers, enabling their assembly into rings or helical structures. However, experimental evidence suggests that these quaternary structures form only at high protein concentrations. Under physiological conditions, ring and helical structures form dynamically, involving monomer-to-multimer transitions tightly linked to DNA binding [10], [8], [9].

Further evidence for ring-based annealing by Rad52 has been recently presented [32]. Our study supports the notion that all five SSAP families can oligomerize, but perhaps the eukaryotic selection for Rad52 reflects a unique property related to multimerization and annealing not shared by bacterial SSAPs.

### 3.2 Defining superfamilies

Our study demonstrates that single-strand annealing has a conserved structural basis, even when sequence conservation is absent. This work exemplifies how large-scale structure prediction can enhance biological knowledge. Since the 1960s, only a few SSAPs have been identified experimentally, while most have been inferred by sequence similarity. The Gene Ontology (GO) term for “DNA double-strand break processing involved in repair via single-strand annealing” lists 145 gene products (accessed on January 3, 2024). However, only four of these annotations are supported by direct assays (GO evidence code: IDA), while most are inferred indirectly (GO evidence code: IBA). The concept of the SSAP superfamily is thus based on a few proteins with direct experimental evidence, with the majority inferred indirectly. This relationship between direct and indirect evidence could be better highlighted in databases like InterPro [20].

Another challenge in defining superfamilies is identifying a common structural motif. The *β*-hairpin and *β*-sheet motifs, known from experimental 3D structures [4], are crucial for DNA-binding function. This knowledge was essential for our study. Without it, large-scale structural searches would be challenging. Recent tools like FoldSeek [33] and large-scale clustering of the AlphaFold database [21], [34] show promise, but they cannot yet establish connections between the five SSAP families. This difficulty arises because regions outside the motifs vary significantly and may contain large disordered segments. Despite this, our study shows that integrating sequence, structure, and functional knowledge can effectively identify these borderline relationships. Applying this approach to all InterPro families remains an open question.

By examining the three experimentally resolved PDB structures—7UJL for Red*β*, 5XRZ for Rad52, and 7UB2 for RecT—Caldwell et al. [16] observed that both Rad52 and RecT use the conserved *β*1-*β*2 motif to wedge into DNA strands. However, structural differences may explain their distinct functional properties.

In Red*β*, a flexible loop (residues 133-138) forms the ‘top’ of the DNA-binding site, modulating access to the binding groove. This flexibility likely contributes to Red*β*'s weaker binding to dsDNA compared to Rad52 [15]. In Rad52, a functionally analogous loop is located at the ‘bottom’ of the DNA-binding site, positioned between the first two *β*-strands of the motif. This arrangement may explain Rad52's tighter interaction with dsDNA.

These distinct structural features, particularly the placement and flexibility of the loop within the DNA-binding site, likely influence the ability of these proteins to bind dsDNA or facilitate annealing. These observations provide a structural basis for differences in DNA-binding properties among SSAP family members and their functional implications.

## 4 Conclusion

In conclusion, our study provides a comprehensive examination of the Rad52 single-strand annealing protein (SSAP) superfamily, offering insights into their evolutionary trajectories and structural diversity. By integrating data from the InterPro database and AlphaFold predictions, we enhanced our understanding of the five SSAP families: Rad52, Erf, Sak3, Red*β*, and RecT.

Our findings support the hypothesis that eukaryotic Rad52 likely evolved from ancestral SSAPs present in both bacterial and archaeal lineages. Despite significant sequence divergence, the structural similarities among SSAPs underscore the functional importance of conserved motifs, particularly the *β*-sheet and *β*-hairpin, in single-strand annealing processes.

Notably, the discovery of a novel oceanic archaeal SSAP that closely resembles human Rad52 highlights the power of integrating structural and sequence data to uncover evolutionary relationships and protein functions. These findings enrich existing knowledge of SSAPs and open new avenues for research, providing a robust framework for further investigations into the evolution and functional mechanisms of these essential proteins.

## 5 Methods

InterPro [20] was accessed on 01.08.2023 for families Rad52 (IPR041247), Erf (PF04404), Sak3 (IPR009425), RecT (IPR004590), and Red*β* (IPR010183). AlphaFold (version 3) [19], [21] structures were retrieved on 02.10.2022. Structures with an average pLDDT confidence score lower than 70% were filtered out. Taxonomic information was extracted from UniProt (accessed 01.09.2023).

The structural similarity was assessed using TM-scores calculated with USAlign [35]. Structures were visualized with PyMOL (version 2.5.0). The dendrograms in Fig. 1 and Fig. S1 were created by hierarchical clustering with average linkage via the Python libraries scikit-bio (version 0.5.7), SciPy (version 1.10.1), and ete3 [36]. They were visualized using iTOL (Interactive Tree of Life). Scatterplots and heatmaps were generated with Seaborn (version 0.12.2) and Matplotlib (version 3.7.1).

For Fig. S1A, sequences were compared using BLAST (version2.8.1+) with the BLOSUM62 substitution matrix. The identity score was determined by multiplying coverage by identity.

The four representatives, A0A842UJM1, A0A843GZ30, L0KZL4, and A0A2D6XBR4, in Fig. S2 were selected as follows: To identify representative structures among the 10,000 single-strand annealing proteins (SSAPs), we applied a hierarchical clustering algorithm based on structural similarity (TM-scores). From the resulting clusters, 12 representative structures were initially selected. These were refined through visual inspection to four structures that best represented the dataset's structural diversity: two from the Rad52 family, one from the Red*β* family, and one from the RecT family. Proteins from the Erf and Sak3 families were omitted due to their structural similarity to Rad52. Scatterplots confirmed that all 10,000 proteins exhibited significant similarity (TM-score) to at least one representative. Algorithm 1 explains the steps, and Fig. 1 shows the positions of these 12 structures and their corresponding 3D structures.

PyMOL was used for Fig. 6 (cealign), Table 3 (secondary structure assignment), and for computing atom clashes (atoms of one monomer within 2Å of atoms of a neighboring monomer). PDB structures 1KN0, 7UB2, and 7UJL served as experimental references.

## Credit authorship contribution statement

**Ali Al-Fatlawi:** Conceived and implemented the study, analysed data, wrote the manuscript. **Md. Ballal Hossen:** Analysed data. **Stella de Paula Lopes:** Analysed data. **A. Francis Stewart:** Conceived the study and wrote the manuscript. **Michael Schroeder:** Conceived and implemented the study, analysed data, wrote the manuscript.

## Declaration of Competing Interest

All co-authors have reviewed and approved the contents of the manuscript, and no conflicts of interest have been declared.

## Data Availability

All data used to generate this work are accessible via the link provided below. This collection includes both raw and generated data. The raw data consists of PDB files for all SSAP proteins and their FASTA sequences. The generated data encompass BLAST results (sequence data), structural alignments (TM scores), taxonomy information, and family memberships from InterPro. Please refer to the following link to access the data. Raw data: ./rawData/[PDBs, Fasta]  Alignment results: ./alignments/  https://sharing.biotec.tu-dresden.de/index.php/s/  vNrJ3aHLSUN6kZE    Video animation for SSAPs across multiple families:  https://youtu.be/7MoSwxtsX-o

## Appendix A Supplementary material

Supplementary material S1. Phylogenetic tree; S2. TM-score distribution; S3. Representative SSAPs; S4 and S5. Models of archaea Rad52 ring; S6. RDM1 structure. Supplementary material

Table S1 TM-scores for all 10280 SSAPs. Table S1

Table S2 TM-scores for unreviewed proteins from UniProt with a TMscore greater than 0.7 to at least one SSAP representative. Table S2
