# Supplementary material for: A structural phylogenetic tree of Rad52 and its annealase superfamily
Source: Comput Struct Biotechnol J. 2024 Dec 24;27:360–8. doi: 10.1016/j.csbj.2024.12.012 (PMC11783212; doi:10.1016/j.csbj.2024.12.012)
Supplement: Supplementary material — S1. Phylogenetic tree; S2. TM-score distribution; S3. Representative SSAPs; S4 and S5. Models of archaea Rad52 ring; S6. RDM1 structure. [file mmc1.pdf]

Supplementary Material

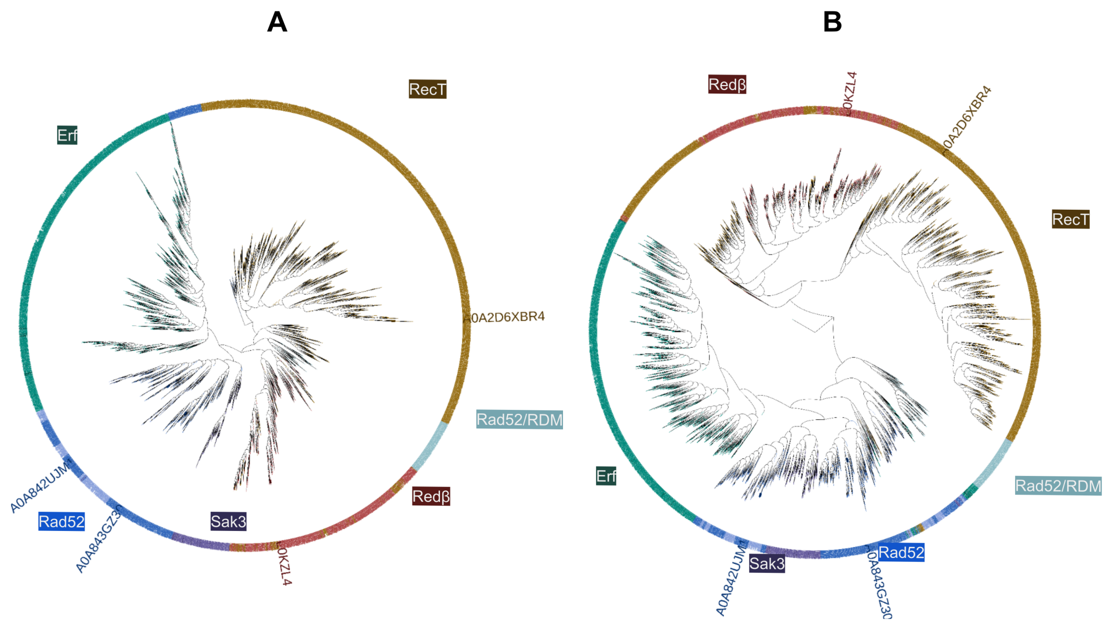

Figure S1: 10280 SSAPs clustered by sequence (A) and by structural (B) similarity. The two trees show a high degree of agreement, as evident from the clustering of major families.

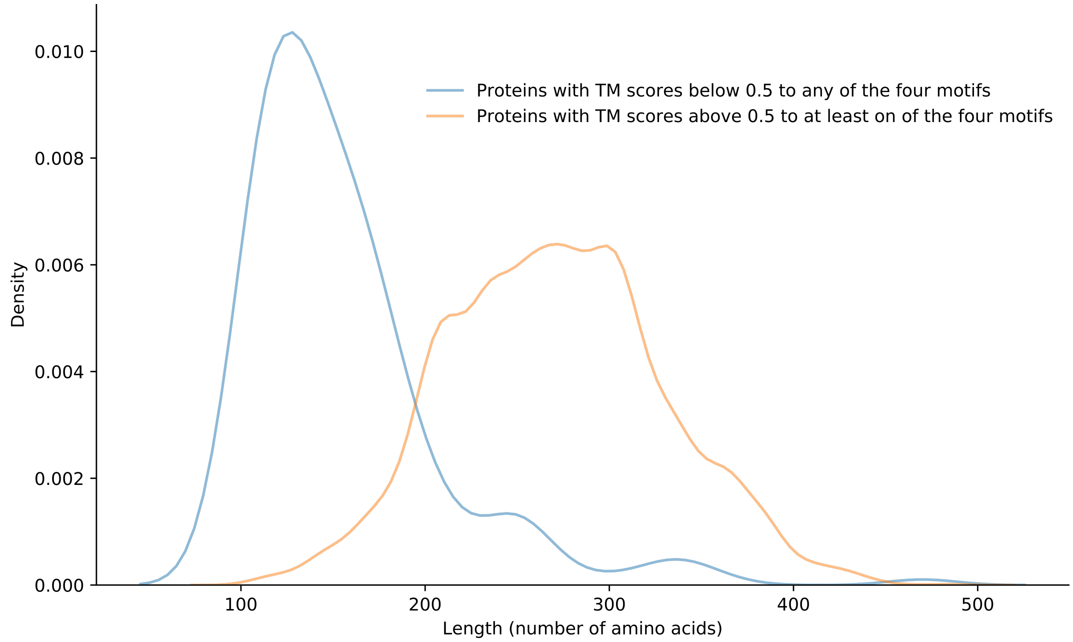

Figure S2: The figure illustrates the distribution of TM-scores for 10280 SSAPs compared to four representative structures. Proteins with TM-scores greater than 0.5 (98%) are highlighted, showing significant structural similarity to at least one representative structure. The 2% of SSAPs with TM-scores below 0.5 are predominantly shorter fragments, with an average sequence length of 160 amino acids, compared to 268 amino acids for the rest of the dataset. These outliers also include a small number of full-length proteins with divergent folds, which may represent edge cases or potential misannotations.

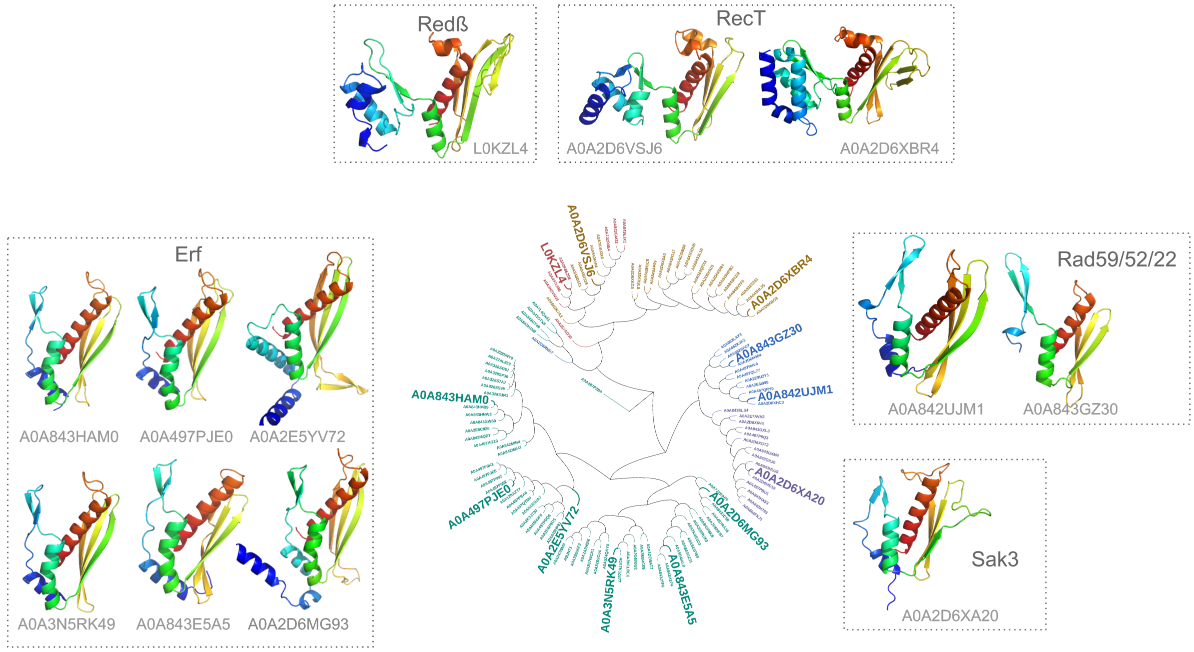

Figure S3: The 12 structures selected by the representative identification algorithm and subjected to visual inspection. From those, we identified the four representatives.

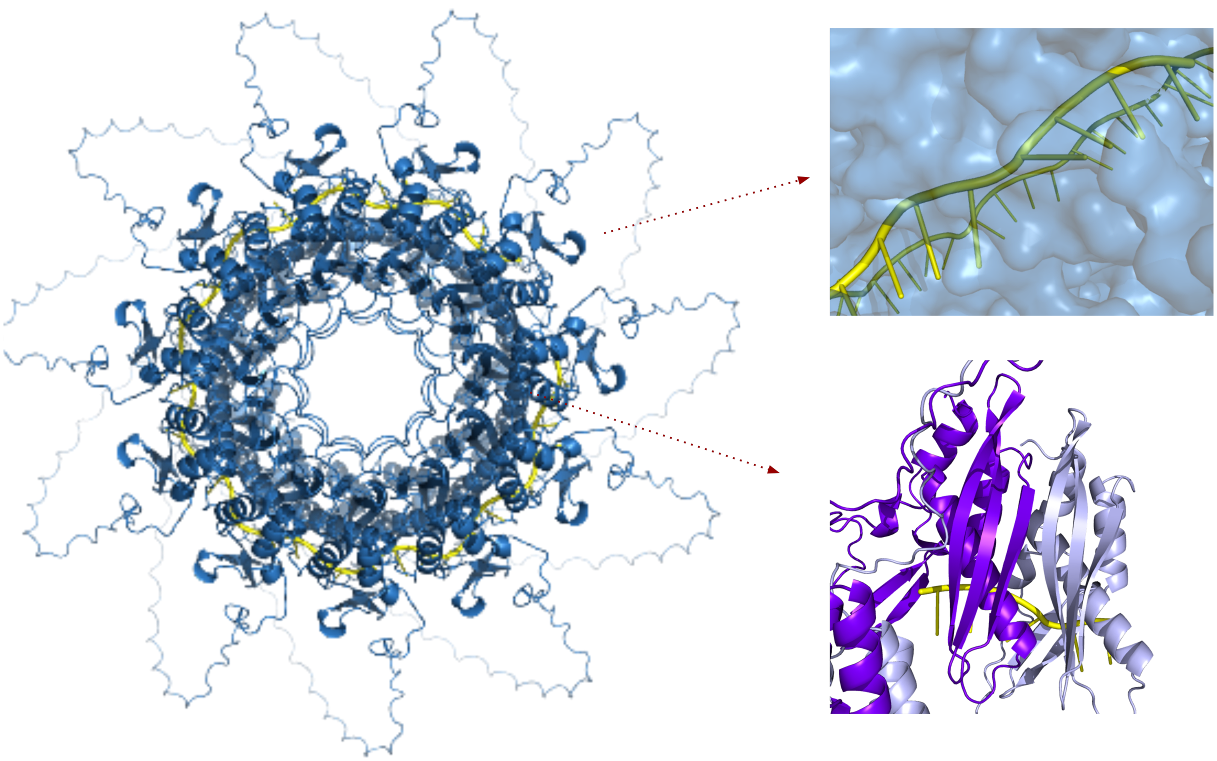

Figure S4: Superposition of the archaeal Rad52 protein (A0A842UJM1) onto the human Rad52 structure (PDB ID: 5XRZ), forming an 11-chain ring visualized with DNA. The untrimmed archaeal structure is shown to illustrate its alignment within the ring. The figure highlights the predicted interaction between the protein (in blue) and the DNA (in yellow), with a zoomed-in view of the chain interaction shown in the lower-right corner.

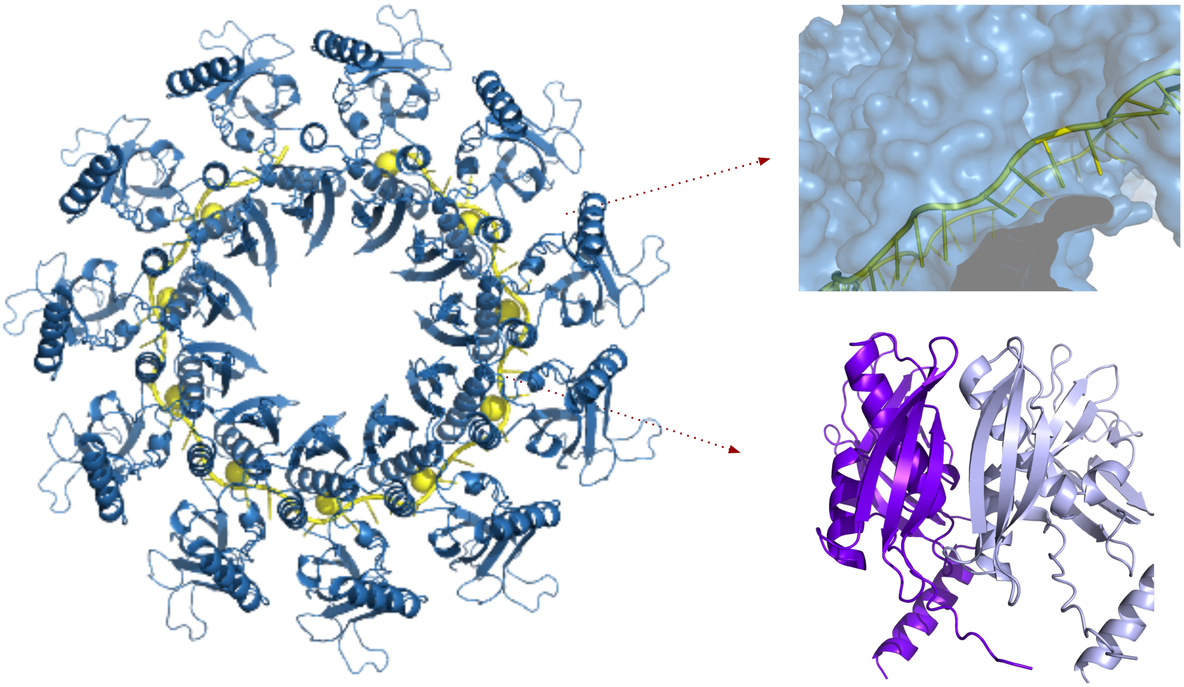

Figure S5: Superposition of the archaeal Rad52 protein (A0A843GZ30) onto the human Rad52 structure (PDB ID: 5XRZ), forming an 11-chain ring visualized with DNA. The untrimmed archaeal structure is shown to illustrate its alignment within the ring. The figure highlights the predicted interaction between the protein (in blue) and the DNA (in yellow), with a zoomed-in view of the chain interaction shown in the lower-right corner.

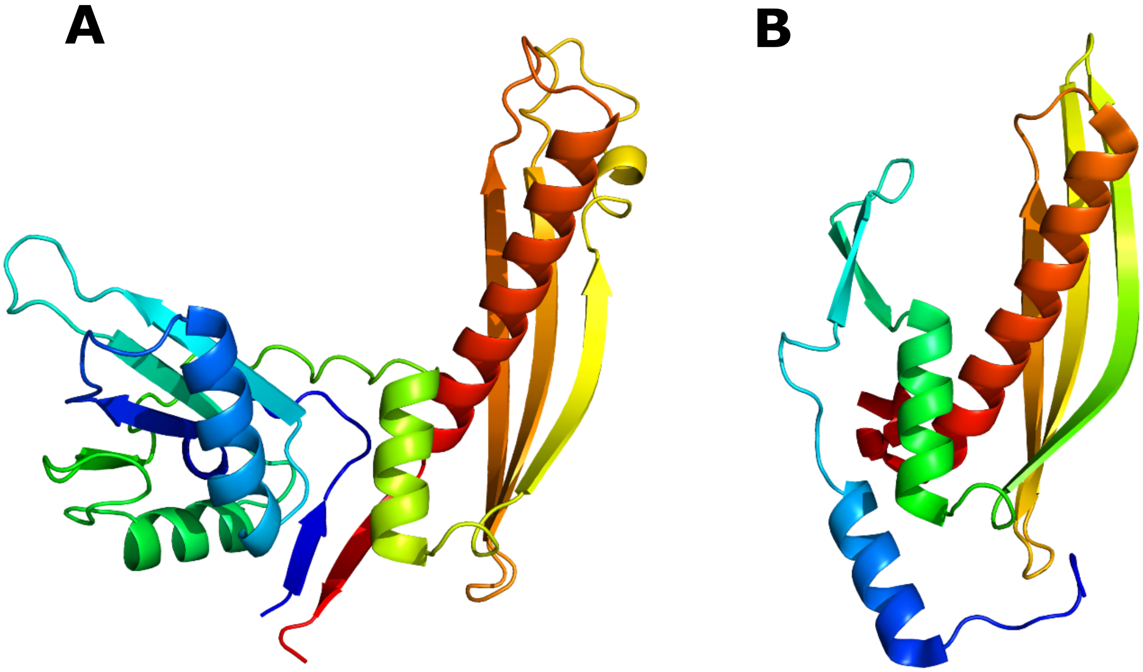

Figure S6: 3D structure of (A) an RDM1 protein predicted with AlphaFold (UniProt: P43351) and (B) a human Rad52 protein determined experimentally (PDB: 5XRZ). Both proteins contain a Rad52-like motif, but in RDM1, the hairpin region of Rad52 is replaced by an RNA-binding motif.

Supplementary tables:

Tables can be accessed through this link: https://sharing.biotec.tu-dresden.de/index.php/s/vozoYURFI3ctWzP

Table S1: TM-scores for all 10280 SSAPs

Table S2: TM-scores for unreviewed proteins from UniProt with a TM-score greater than 0.7 to at least one SSAP representative.

Algorithm 1 Phylogenetic Tree Clustering and Representative Identification

Require: Phylogenetic tree in Newick format, pairwise distance matrix, metadata.

Ensure: Cluster assignments and representative nodes for each cluster.

```
Cluster the Tree:
Load the phylogenetic tree from the Newick file.
Apply the TreeCluster algorithm [37] to partition the tree:
for each cluster identified do
    Extract Nodes:
    Retrieve all nodes belonging to the current cluster.
    Calculate a Linkage Matrix:
    Compute the pairwise distances for nodes within the cluster.
    Apply hierarchical clustering using the average method to compute the
    linkage matrix.
    Build a Newick Tree:
    Construct a tree structure from the linkage matrix:
        • Treat each node as a leaf.
        • Iteratively merge nodes based on the linkage matrix distances.
        • Convert the final tree structure to Newick format.
    Find the Common Ancestor:
    Parse the constructed Newick tree.
    Identify the common ancestor of all leaf nodes in the cluster.
    Select a Representative Node:
    Compute similarity scores between the common ancestor and each leaf.
    Choose the leaf with the highest similarity score as the representative
    for the cluster.
end for
return Cluster assignments and representative nodes.
```
